# Corticosteroid injection for pyogenic granuloma of the tongue in a rare resembling ulcerative malignancy: a case report

**DOI:** 10.1186/s12903-025-06889-0

**Published:** 2025-09-29

**Authors:** Yinchun Zhang, Qinghua Mao, Yinyan Wang, Fei Sun, Chuanxia Liu

**Affiliations:** 1 Shaoxing Stomatological Hospital, Zhejiang Shaoxing, 312000 China; 2 Stomatology Hospital, School of Stomatology, Zhejiang University School of Medicine, Clinical Research Center for Oral Diseases of Zhejiang Province, Key Laboratory of Oral Biomedical Research of Zhejiang Province, Cancer Center of Zhejiang University, Stomatology Hospital, School of Stomatology, Zhejiang University School of Medicine, Hangzhou, Zhejiang 310006 China

**Keywords:** Pyogenic granuloma, Ulcerative mass, Tongue malignancy, Corticosteroid injection, Case report

## Abstract

**Background:**

Pyogenic granuloma (PG) is a benign vasoproliferative lesion that usually occurs in the skin And mucosa. The clinical manifestations of PG are rapid growth, pedicled or broad base of a single red smooth papule, easily bleeding with minor lesions. Most of the oral lesions occur at the gingiva, And the extra-gingival lesions is less common, and the ulcerative type is even rarer. We present a case of ulcerative lesion located at the lateral margin of the tongue with a peripheral bulge for more than 5 years. It was initially suspected to be tongue cancer and was confirmed to be a PG by microscopic examination, and the ulcer healed and the protrusion subsided after topical steroid corticosteroid injection.

**Case presentation:**

A 75-year-old woman presented with An ulcer on the right tongue for more than 5 years. Clinical examination revealed a prominence seen on the mid-posterior part of the lateral margin of the right tongue with a tough texture and an ulcerated surface visible in the center. The Lingual cusps of the right lower first And second molars opposite the ulcer was sharp. Interestingly, the patient had undergone partial gastrointestinal resection because of gastric carcinoma And had been on antidepressants for 4 years. Hematoxylin-eosin (HE) staining was suggestive of vascular and vascular endothelial cell hyperplasia in the lamina propria of the ulcer surface, and the pathologic diagnosis was PG. Two injections of *triamcinolone acetonide acetate* (mixed with *lidocaine hydrochloride*) were administered at 2-week intervals, combined with topical *bovine basic fibroblast growth factor* (bFGF) and *cetylpyridinium chloride/compound chlorhexidine gargle.* Complete healing was observed at 4 weeks, with no recurrence confirmed by telephone follow-up at 6-month and1-year post-treatment.

**Discussion:**

Extragingival PGs in the mouth are relatively rare, and longstanding ulcerative protrusions occurring on the lateral margins of the tongue are often recognized as ulcerative tumors, and pathology is necessary to distinguish malignant tumors from PGs. Trauma, altered hormone levels, and medications can all contribute to the development of PGs, and we hypothesize that the sharp dental cusps, altered estrogen secretion, long-term use of antidepressant medications, and partial gastrointestinal resection may contributed to the development of PG in this woman. The conventional treatment for PG is surgical resection. In this case, good therapeutic effect was achieved after using multi-point topical injection of corticosteroid, combined with wet compress, mouthwash, and medication application, which provides clinical basis for the selection of non-surgical treatment for PG.

## Background

Pyogenic granuloma (PG) is an acquired, benign, vasoproliferative lesion that usually occurs in the skin or mucosa. When the tissue is irritated, it can lead to proliferation of granulation tissue within the skin or mucosa. Although it often manifests as a tumor-like growth, its essence is nonneoplastic [1]. In 1904, Hartzell was the first to use the term “pyogenic granuloma” or “granuloma pyogenicum” [2]. However, the term “pyogenic” is often misunderstood, but in fact it is not a suppurative inflammation caused by a pyogenic bacterial infection, and the lesion does not contain pus [3]. Although PG is a relatively common skin lesion, its incidence in the oral cavity is relatively low [4], and about 75% of PG occurring in the oral mucosa occurs in the gingiva [5], with extragingival PG being less common. Clinically, PG presents as a relatively soft-textured mass, which may have a smooth or lobulated surface and often ranges in diameter from a few millimeters to several centimeters [6]. However, when PG presents as a long-standing ulcerative protrusion on the lateral margins of the tongue – as in the case we report – its clinical appearance can closely mimic that of oral malignancy. This potential for misdiagnosis underscores the critical importance of accurate differentiation.

Oral cancer is the sixth most common cancer in the world, posing a serious threat to human health, with an incidence rate of about 1/275,000 [7]. About 90% of oral cancers are oral squamous cell carcinomas (OSCC), with a higher incidence in males than in females, about 2:1 [8]. Notably, the tongue, especially its lateral borders and ventral surfaces, is a predilection site for OSCC [9]. The typical presentation of OSCC includes persistent ulceration, induration (increased hardness), necrosis, bleeding, and tenderness [10–12]. Ulcerative lesions are the most common manifestation of OSCC, often developing as the disease progresses; these ulcers frequently exhibit irregular shapes with raised, rolled, and indurated margins upon palpation [12, 13]. Therefore, any chronic, non-healing ulcer in the oral cavity, especially in high-risk locations like the tongue border, warrants thorough investigation to exclude malignancy [13]. However, it is recognized that ulcerative PG may present with similar clinical manifestations. Thus, histopathological examination is required to determine the exact diagnosis.

We present a case in which a malignant tumor of the tongue was suspected based on the initial clinical presentation, however, the diagnosis of PG was confirmed by histopathological examination, and the lesion disappeared after intralesional injection of corticosteroid hormones, in conjunction with topical medication. At the same time, we briefly reviewed the clinical presentation and pathology of PG and oral cancer, and analyzed the possible risk causes for the induction of PG in this patient based on her systemic condition, local irritation, and history of medication use.

## Case presentation

**Fig. 1:**
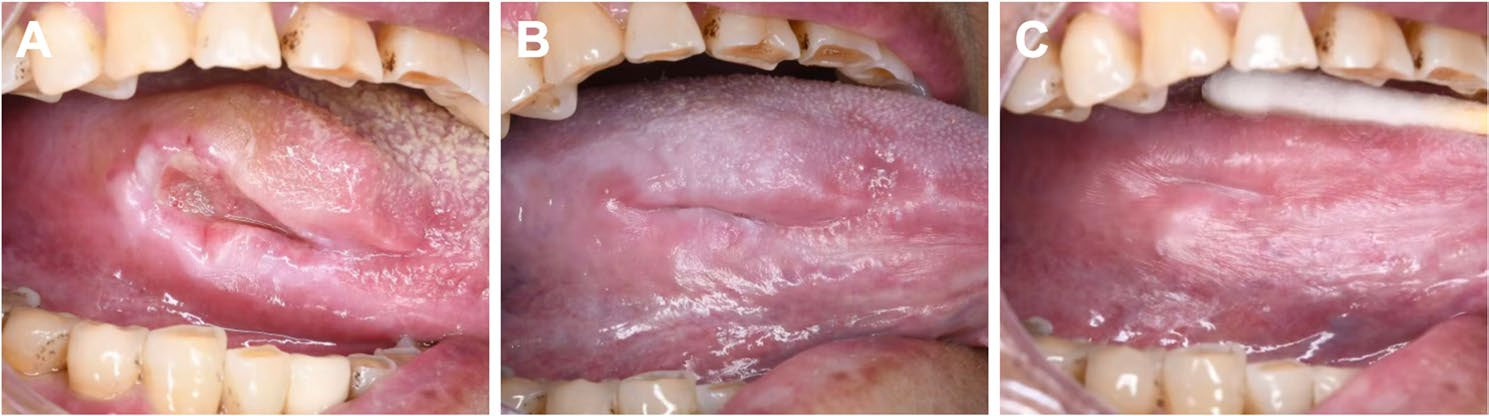
Clinical manifestations of therapeutic effects. **A** Before treatment. **B** 2 weeks after the first corticosteroid injection with the use of topical medications (2 weeks follow-up). **C** 2 weeks after the second corticosteroid injection with the use of topical medications (4 weeks follow-up)

A 75-year-old woman presented to the outpatient clinic of the department of oral medicine, Stomatology Hospital, School of Stomatology, Zhejiang University School of Medicine after suffering from a tongue ulcer And constant pain for more than 5 years. She had used mouthwash and oral antibiotics for many times, but the ulcer and recurrent pain was never healed. She denied having bitten through the area prior to the initial appearance of the ulcer. She also told us that she had undergone a partial gastrointestinal resection for her gastric carcinoma many years ago and that she had started taking antidepressant medication for depression four years ago, but did not tell us the exact drug. Allergies and history of other diseases and medications were denied. The patient also denied any history of tobacco use or alcohol consumption.The physical examination revealed a protruding mass of about 3cm×3 cm×2 cm in size at the mid-posterior part of the right Lingual margin, with relatively clear borders, average mobility, peripheral elevation, And tough texture. An ulcer of about 1.5 cm×0.8 cm was visible on the surface of the center of the mass, with no redness around the ulcer (Fig. 1A). We also examined the teeth in the area adjacent to the mass and found that her right maxillary first and second molars were missing, the lingual cusps of the right mandibular first and second molars were sharp, and there was grade Ⅲ° mobility of the right mandibular second molar.

**Fig. 2:**
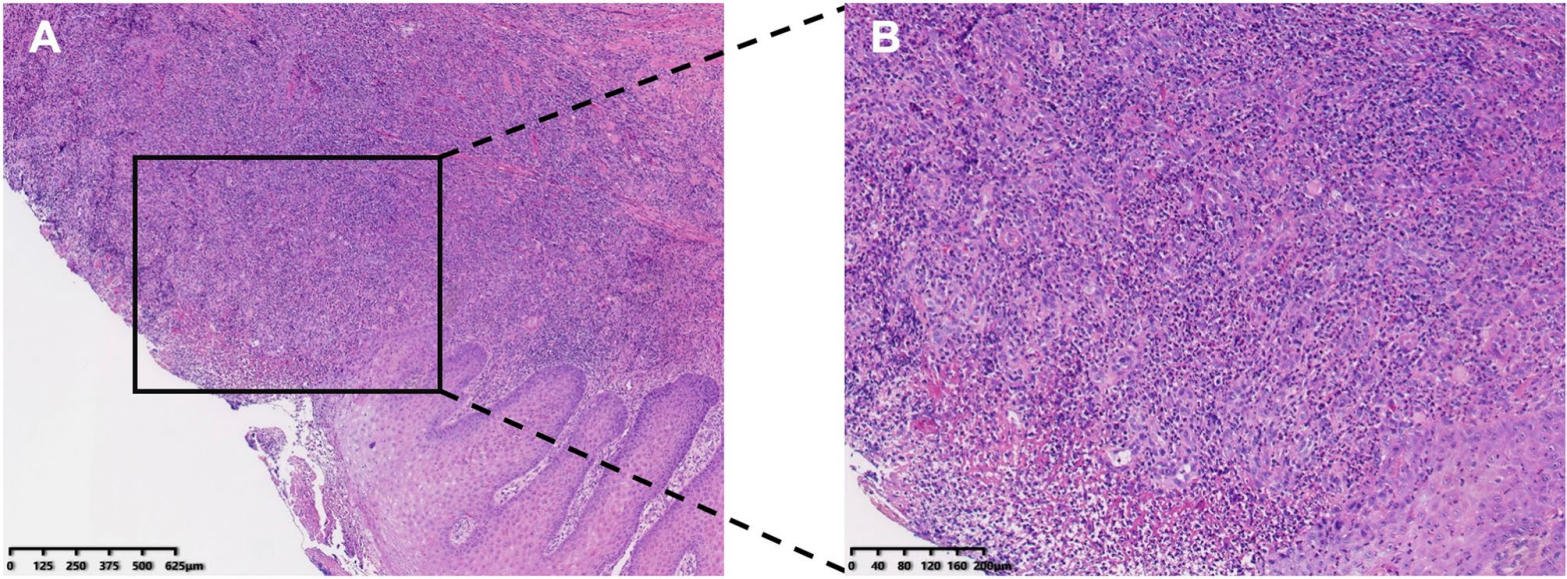
Histopathology images. **A** 4× magnification. **B **10× magnification

Based on the patient’s complaints and clinical manifestations, we suspected that the mass on the right side of the tongue might be a malignant lesion. After obtaining the patient’s informed consent and ruling out contraindications to biopsy surgery, the sharp edges of the teeth were polished to make them smooth, and part of the tissue at the border of the tongue ulcer was cut out in preparation for a pathologic examination. Pathology showed ulceration of the mucosal surface of the dorsum of the tongue, hyperplasia of blood vessels and vascular endothelial cells in the lamina propria, and infiltration of neutrophils, eosinophils, lymphocytes, and plasma cells, which led to the diagnosis of “pyogenic granuloma” (Fig. 2).

The therapeutic protocol was performed as follows: (1) Drug preparation and injection: *triamcinolone acetonide acetate* (5 mL:50 mg) was mixed with 2% *lidocaine hydrochloride* (5 mL:0.1 g) in a 1:1 ratio using a sterile 5 mL syringe; using a 27-gauge sterile injection needle, the mixture (total volume 0.8 mL) was injected at four quadrants around the ulcer base (3, 6, 9, And 12 o’clock positions), each injection site received 0.2 mL of the suspension (containing 1 mg *triamcinolone* And 2 mg *lidocaine* per site); the injection depth was in the submucosal layer; before injection, aspiration should be performed to prevent intravascular injection; after injection, it should be compressed for 2 min to prevent hematoma formation. (2) Topical application: Apply wet compress treatment locally (*triamcinolone acetonide acetate* solution diluted to 2 mg/mL with saline), rinse *cetylpyridinium chloride gargle* daily (15 mL/time, gargle for 1 min, three times a day), and apply *bovine bFGF gel* (21000 IU: 5 g) locally once a day at a dosage of 300 IU per square centimeter.

At the 2-week follow-up visit, the patient felt a recent reduction in pain and a significant reduction in the size of the lesion was observed (Fig. 1B). The ulcer size was reduced to about 0.5 cm × 0.1 cm with a slightly elevated margin. A mixture of *lidocaine hydrochloride* solution and *triamcinolone acetonide acetate* suspension was again injected intralesionally. The patient was told to continue to use topical external medication, and to prevent bacterial dysbiosis caused by prolonged use of the same gargle solution, we replaced the *Cetylpyridinium Chloride* with the c*ompound chlorhexidine gargle*. The topical application protocol was repeated at the 2-week follow-up visit.

After another 2 weeks, the patient had no pain, and examination revealed that the ulcer surface had healed, with a linear scar visible in the center, and the marginal tissue elevation was significantly less than before, only slightly protruding from the mucosal surface, with a soft texture (Fig. 1C).

The patient was scheduled for clinical review at 3, 6, And 12 months post-treatment. However, the patient was unable to attend the scheduled reviews due to relocation to Another city. At the 6 months And 1 year after treatment, a telephone follow-up was conducted. The patient reported complete resolution of pain and no recurrence of the lesion, indicating sustained therapeutic efficacy.

## Discussion and conclusion

Ulcers that do not heal for a long time in the mouth have a risk of cancer, especially those located in high-risk areas such as lingual border. They need to be differentiated from oral cancer, and pathology is the gold standard for differentiation. PG is usually difficult to differentiate from oral malignancy when ulceration is present on the surface for a long time. As a limited lesion, PG usually has clear borders and regular shape. Because it is composed of mainly neoplastic capillaries and endothelial cells, it is relatively soft in texture and bleeds easily upon irritation [14]. Oral cancer, on the other hand, will become slightly harder in texture as the cancer cells proliferate and infiltrate the surrounding tissues [10]. The reason for the tougher texture of the PG in this case may be due to the fact that after repeated mechanical stimulation, the tissue is repeatedly in the process of injury and repair, resulting in a proliferative buildup of the fibrous tissues involved, which may be the reason for the lack of significant bleeding of the PG under prolonged sharp-tip stimulation in this case. PG is usually bright red or dark red due to its abundant capillaries. In oral malignant tumors, the tumor cells grow fast, And local tissue necrosis occurs due to insufficient blood supply on the surface of the mass and appears grayish-white. Oral cancer is mostly OSCC. Notably, this female patient presented with a painful ulcerative mass on the tongue interfering with mastication, which had persisted for five years. However, the tongue remained fully mobile, and physical examination revealed no enlarged cervical lymph nodes. These clinical findings are less suggestive of malignancy. OSCC in high-risk sites such as the lateral tongue border typically exhibits aggressive behavior. A study of 171 patients with early-stage tongue OSCC reported lymph node metastasis in 40 cases (23.4%) [15]. Ahmed SQ et al. reported in their study of 78 early-stage tongue OSCC patients that cervical lymph node metastasis occurred in 69% of cases with tumor thickness > 5 mm, whereas no metastasis was observed in patients with tumors < 5 mm in thickness [16]. Pathologically, PG consists of lobular proliferative granulation tissue forming a rich vascular network, infiltration of scattered inflammatory cells, and an increased fibrin layer within the epithelium is seen within lesions that have ulcerated [17]. In contrast, disorganized squamous epithelial cells with varying degrees of differentiation and significant cellular anisotropy can be observed in OSCC. Certain PGs share similar clinical manifestations with OSCC, so histopathologic examination is important in determining the nature of both.

PG is often caused by traumatic injury, inflammation, hormonal changes, or medications [14]. This patient had sharp dental cusps in the lesion counterpart, which may have been the most important factor contributing to this patient’s tongue PG. Physical friction or chemical irritation causes the release of a variety of endogenous substances and angiogenic factors in the corresponding areas of the oral mucosa resulting in a disturbed vascular distribution in the lesion area [18]. Therefore, we speculate that long-term and sustained sharp cusps stimulation can keep tongue lesions in an inflammatory state for a long time, breaking the normal damage/repair mechanism. This sustained inflammatory environment will continuously release growth factors in local tissues, which will further stimulate the growth of blood vessels and fibrous tissues. A study by Yuan et al. found that several important angiomorphogenetic factors have higher levels in PG than in normal tissues, suggesting that PG may result from an imbalance between these angiomorphogenetic factors [19]. Therefore, we immediately regulated the grinding of the sharp cusps of the teeth as soon as this risk factor was detected.

In this case, the patient was at an older age and we hypothesized that the PG might also be related to its altered estrogen secretion levels. At this stage, ovarian function declines, resulting in a significant decrease in estrogen. Estrogen has an important protective effect on the oral mucosa, and under normal conditions, estrogen promotes the proliferation and differentiation of oral mucosal cells and maintains the thickness and elasticity of the mucosa. With decreased estrogen, the self-repairing ability of the oral mucosa decreases [20]. Therefore, when the oral mucosa is damaged, the repair process may be abnormal and tend to trigger an inflammatory response, which can increase the risk of PG. However, PG is more common in women during pregnancy, when the level of estrogen secretion increases significantly. Existing studies suggest that the increase in estrogen may have an effect on the microcirculatory system, stimulating angiogenic factor production, increased vascular permeability, and vascular proliferation, which may trigger PG [17, 21]. Therefore, whether lack of estrogen in women can trigger or induce PG still requires further study.

This woman has been taking antidepressants for a long time, and the main antidepressants currently in use are Selective Serotonin Reuptake Inhibitors (SSRIs), and there have been no reports of antidepressant-induced PG in the literature. But some scholars have also discovered drug-related PG, and *carbamazepine* may stimulate the release of angiogenic factors through inflammatory processes [22]. *Levothyroxine*, through its promotion of angiogenesis and proliferation, may be a possible cause of PG occurrence [23]. The use of SSRIs can lead to an increase in inflammatory markers in vivo, and cytokine expression levels of Tumor Necrosis Factor-α (TNF-α) and Interferon-γ (IFN-γ) were increased in depressed patients taking SSRIs [24]. We hypothesized that the patient’s use of antidepressants may lead to altered levels of some of these inflammatory factors, which in turn affects the permeability of the vascular endothelium, which may act as a trigger for the production of PG.

This patient has a history of gastric carcinoma, which increases the incidence of oral cancer. On the other hand, the patient had also undergone a partial gastrointestinal resection, which is associated with impaired absorption of nutrients, and the intake of some nutrients plays an important role in maintaining the integrity and function of the oral mucosa. For example, vitamin B_12_ deficiency interferes with the normal metabolism and repair of the oral mucosa [25]. In addition, there is a close correlation between the microbial communities of the gastrointestinal tract and the oral cavity [26]. Gastrointestinal resection surgery disrupts the gastrointestinal microbiotal community, which in turn has a certain impact on the oral microbial community. The imbalance of oral microbiota makes patients more susceptible to inflammatory responses after local stimulation.

Oral PG is prone to recurrence, with a recurrence rate of 15%. The recurrence rate of PG in the gingiva is much higher than that in other parts of the oral mucosa [27]. Given the high recurrence risk associated with PG, long-term surveillance remains imperative. Although the 6-month and 1-year telephone follow-up confirmed no recurrence, this approach has inherent limitations: inability to visually inspect the lesion site; absence of palpation for submucosal changes; and potential bias in patient self-reported symptoms.

The conventional treatment is surgical resection, and minimally invasive treatments include laser, corticosteroid injections, cryotherapy, and sclerotherapy [28]. After the patient was identified as a PG, we administered a mixture of *triamcinolone acetonide acetate* and *lidocaine hydrochloride* to the patient for local multipoint injection. Considering the patient’s poor tolerance, performing non-surgical treatment can avoid secondary incisions in the short-term period after the patient’s biopsy and also reduce the trauma during surgery. Mixing with *lidocaine hydrochloride* in equal proportions reduces the concentration of triamcinolone acetonide while simultaneously alleviating injection site pain. As a corticosteroid, triamcinolone inhibits the production of key inflammatory mediators—including prostaglandins, leukotrienes, and cytokines—and significantly decreases vascular permeability [29]. Corticosteroids were found to reduce vascular endothelial growth factor (VEGF) expression and angiogenic potential, which may account for the effective treatment of oral PG with corticosteroids [30]. Parisi et al. reported for the first time the use of corticosteroids for the treatment of oral PG. They administered four times injections over 9 weeks to a patient with PG accompanied by satellite lesions, and observed improvement in the lesions at each visit [31]. Amr Bugshan et al. injected corticosteroids into five different sites of palatal PG lesions in conjunction with topical application of 0.05% *clobetasol propionate ointment* for 2 weeks, And the lesions had completely disappeared at the 3-week follow-up [32]. Additionally, adjunctive wet compress application of triamcinolone acetonide enhances the local anti-inflammatory effects of corticosteroid therapy.

The ulcerated surface of the patient’s PG lesion is highly susceptible to colonization and infection by abundant oral bacteria. These microorganisms and their metabolites act as potent inflammatory stimuli, exacerbating local inflammatory responses. The *compound chlorhexidine gargle* contains both chlorhexidine and metronidazole. As a cationic agent, chlorhexidine electrostatically binds to anionic bacterial cell walls, disrupting membrane permeability and inducing bacterial death through leakage of intracellular components [33]. Metronidazole’s nitro group undergoes anaerobic reduction to cytotoxic intermediates that disrupt bacterial DNA structure and inhibit nucleic acid synthesis, culminating in cell death. *Cetylpyridinium chloride*, a quaternary ammonium cationic surfactant, exerts antimicrobial effects via electrostatic adsorption of its cationic moieties to anionic microbial membranes. This competitive displacement disrupts membrane integrity and induces lysis in bacteria and fungi [34]. C*ompound chlorhexidine gargle* or *cetylpyridinium chloride* mouthwashes facilitate ulcer healing in PGs by reducing local irritants and bacterial load, thereby optimizing the oral microenvironment for tissue repair.

*Bovine bFGF gel* contains a substantial amount of bFGF, a potent mitogen for fibroblasts and a significant angiogenic factor. At ulcer sites, it markedly stimulates the proliferation and migration of nearby fibroblasts and vascular endothelial cells into the wound, promoting tissue repair and healing [35]. However, in cancerous ulcers, bFGF can directly stimulate the proliferation of cancer cells, accelerating tumor growth and infiltration [36, 37]. We previously confirmed the absence of cancer cells through histopathological examination before utilizing *bovine bFGF gel* to promote ulcer healing.

In conclusion, this case highlights the need to differentiate ulcerative PGs from oral malignancies, prioritize the provision of non-surgical treatment options with a clear pathologic diagnosis is more acceptable for older patients, And provides insights for further clinical understanding of extragingival PGs. Despite relocation-related follow-up limitations, 1-year remission supports the efficacy. Long-term monitoring remains crucial, and future research can standardize remote assessment protocols for such cases.

## Data Availability

No datasets were generated or analysed during the current study.

## Abbreviations

PG: Pyogenic Granuloma
HE: Hematoxylin-Eosin
OSCC: Oral Squamous Cell Carcinomas
TNF-α: Tumor Necrosis Factor-α
IFN-γ: Interferon-γ
VEGF: Vascular Endothelial Growth Factor
bFGF: basic fibroblast growth factor
